# Methods for identification of the opportunistic gut mycobiome from colorectal adenocarcinoma biopsy tissues

**DOI:** 10.1016/j.mex.2024.102623

**Published:** 2024-02-23

**Authors:** Aisyah Yunus, Norfilza Mohd Mokhtar, Raja Affendi Raja Ali, Siti Maryam Ahmad Kendong, Hajar Fauzan Ahmad

**Affiliations:** a Faculty of Industrial Sciences and Technology, Universiti Malaysia Pahang Al-Sultan Abdullah (UMPSA), Lebuhraya Tun Razak, 26300 Gambang, Pahang, Malaysia; b Department of Physiology, Faculty of Medicine, Universiti Kebangsaan Malaysia (UKM), 56000 Kuala Lumpur, Malaysia; c Gut Research Group, Faculty of Medicine, Universiti Kebangsaan Malaysia, 56000 Kuala Lumpur, Malaysia; d School of Medical and Life Sciences, Sunway University, 47500 Subang Jaya, Selangor, Malaysia; e Department of Basic Medical Sciences, Faculty of Medicine and Health Sciences, Universiti Malaysia Sarawak, 94300 Kota Samarahan, Sarawak, Malaysia

**Keywords:** Identification of opportunistic gut fungal pathogens in biopsies of CRC patients, Gut mycobiome, Fungal microbiome, Colorectal cancer, Cancer biomarkers, Polymerase chain reaction, Next-generation sequencing

## Abstract

Colorectal cancer poses a significant threat to global health, necessitating the development of effective early detection techniques. However, the potential of the fungal microbiome as a putative biomarker for the detection of colorectal adenocarcinoma has not been extensively explored. We analyzed the viability of implementing the fungal mycobiome for this purpose. Biopsies were collected from cancer and polyp patients. The total genomic DNA was extracted from the biopsy samples by utilizing a comprehensive kit to ensure optimal microbial DNA recovery. To characterize the composition and diversity of the fungal mycobiome, high-throughput amplicon sequencing targeting the internal transcribed spacer 1 (ITS1) region was proposed. A comparative analysis revealed discrete fungal profiles among the diseased groups. Here, we also proposed pipelines based on a predictive model using statistical and machine learning algorithms to accurately differentiate colorectal adenocarcinoma and polyp patients from normal individuals. These findings suggest the utility of gut mycobiome as biomarkers for the detection of colorectal adenocarcinoma. Expanding our understanding of the role of the gut mycobiome in disease detection creates novel opportunities for early intervention and personalized therapeutic strategies for colorectal cancer.

• Detailed method to identify the gut mycobiome in colorectal cancer patients using ITS-specific amplicon sequencing.
• Application of machine learning algorithms to the identification of potential mycobiome biomarkers for non-invasive colorectal cancer screening.
• Contribution to the advancement of innovative colorectal cancer diagnostic methods and targeted therapies by applying gut mycobiome knowledge.

## Graphical abstract

**Figure:**
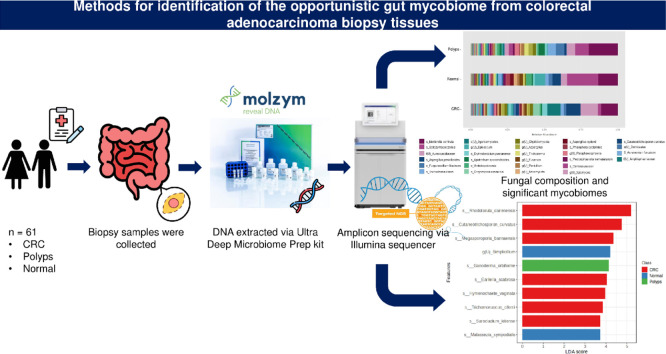

Specifications Table

**Table:** 

Subject area:	Biochemistry, Genetics and Molecular Biology
More specific subject area:	Molecular Identification of Fungal Mycobiome
Name of your method:	Identification of opportunistic gut fungal pathogens in biopsies of CRC patients
Name and reference of original method:	Yunus A et al. (2023) IDDF2023-ABS-0301 Pathogenic fungi of rhodotorula dairenensis is linked with colorectal cancer patients in malaysia. Gut, 72(Suppl 1), A132–A132 http://dx.doi.org/10.1136/gutjnl-2023-IDDF.116[1]
Resource availability:	NCBI Sequence Read Archive (SRA) data Equipment: Illumina NovaSEQ6000 (2 × 150 bp configuration) Software: QIIME 2, MicrobiomeAnalyst 2.0

## Method details

### Background

Colorectal cancer (CRC) is a common and life-threatening disease [2] that requires the development of effective diagnostic and therapeutic strategies [3]. The gut microbiome, including the mycobiome (fungal community), has emerged as a potential contributor to CRC pathogenesis [4]. Nonetheless, the precise role of the mycobiome in CRC, particularly in those without a hereditary predisposition, remains essentially unexplored. Understanding the relationship between the gut mycobiome and CRC may result in the development of noninvasive screening methods for early detection. When opposed to stool samples, microbiome research based on biopsy samples provides unique problems since biopsies include fewer microbial cells but are rich in human genomes, making it more challenging to identify and analyze microbial communities [5,6]. Additionally, the biopsy samples may introduce significant biases toward the detection of particular microorganisms, such as those that stick to the mucosal wall of the colon [7]. These obstacles necessitate the use of advanced sequencing techniques and bioinformatics tools in order to surmount limitations and obtain meaningful insights from microbiome studies based on biopsy samples [8].

The purpose of this study is to investigate the potential of the gut mycobiome as a noninvasive CRC screening tool. To accomplish this, amplicon sequencing focusing on the ITS1 region is proposed to analyze the taxonomic profiles of fungal communities in the gastrointestinal contents of CRC patients [1]. The sequenced data can be effectively interpreted using machine learning algorithms to identify prospective mycobiome biomarkers associated with CRC. In addition, the incorporation of bioinformatics tools can assist in uncovering the functional pathways implicated in the interaction between mycobiome and CRC [9]. A deeper understanding of the role of the gut mycobiome in CRC could pave the way for the development of novel diagnostic strategies based on the fungi community and targeted therapies related to fungal infections. This study aimed to elucidate the complex relationship between the gut mycobiome and CRC, providing valuable insights for future studies and clinical applications.

### Sample collection and processing

Over a period of 18 months, patients with CRC were recruited from Hospital Canselor Tuanku Muhriz UKM, also known as the Universiti Kebangsaan Malaysia Medical Centre. All applicable ethical standards and guidelines were followed to protect the rights of participants. The ongoing sample collection for the study entitled “Exploring Diet, Physical Activities, and the Host's Interaction with the Colonic Mucosal Microbiota Population in Young Colorectal Cancer” has been registered and granted Ethical Clearance (UKMSPPI- BO02).

Patients with polyps and normal patients who did not have a prior history of illness were asked for their informed consent in order to participate in this study. During the colonoscopy procedures, authorized medical personnel performed the collection of polyp samples. Tumor samples were collected using collection tubes containing RNAlater solution (ThermoFisher Scientific, USA) from patients who underwent colorectal anterior resection and stored at −80 °C.

### Materials, reagents, and solutions

In this study, the Ultra Deep Microbiome Prep (Brand: Molzym, Germany) kit was used in the DNA extraction, and it consists of Kit 1 – Buffers and Consumables (storage temperature: 18 to 25 °C) and Kit 2 – Enzymes and Reagents (storage temperature: −15 to −25 °C). Kit 1 includes essential buffers such as buffer CM, DB1, RS, RL, RP, CS, AB, WB, TSB, and PKB, as well as consumables such as sample tubes, spin columns, collection tubes, and elution tubes. Furthermore, Kit 2 consists of enzymes such as MolDNase B, and Proteinase K, along with the reagents BugLysis, and *β-mercaptoethanol*. Additionally, this study utilized 70% ethanol, deionized water, distilled water, agarose gel, 40xTris-acetate-EDTA (TAE) buffer, PCR reaction mixture, Diamond™ Nucleic Acid Dye (Promega, Germany), DNA ladder, and 6x loading dye.

### Procedure for microbial DNA extraction from biopsy samples

The microbial DNA was extracted from biopsy samples using Ultra-Deep Microbiome Prep (Brand: Molzym, Germany) kit, a comprehensive kit for ultra-sensitive identification of a broad range of bacteria and fungi from a variety of sample types including biopsies, blood, primary body fluids, human and animal tissues, as well as biofilms [10]. The following are the finalized protocols, which were modified slightly to assure the efficient dissolution of biopsies in the solution mixture, as well as to enhance the DNA quality and quantity for the analysis.

**A. Pre-treatment of tissue biopsy samples**

• Tissue biopsies were sampled and transported under conditions that avoided contamination.
• 180 µL of buffer PKB (Kit 1) was pipetted into a sample tube (Kit 1).
• The sample was transferred to sterile support, e.g., a petri dish, and cut into small pieces (∼0.5 × 0.5 cm) using a sterile scalpel.
• The cut sample was then transferred to the sample tube filled with buffer PKB, and 20 µL of Proteinase K (Kit 2) was added.
• The mixture was vortexed for 15 s and incubated for 1 hour at 56 °C and 1000 rpm using a thermomixer.
• The transport solution was filled up to 1 mL if available, or with buffer TSB, using the measuring line of the tube.

**B. Sample pre-treatment and DNA isolation procedure**

• For each sample, 250 µL of buffer CM was added to the sample tube and vortexed for 15 s.
• The sample was left to stand at room temperature (+18 to +25 °C) for 5 min.
• Following this, the sample was briefly centrifuged, and 250 µL of buffer DB1 was added to the sample tube.
• 10 µL of MolDNase B (Kit 2) was added and vortexed for 15 s.
• The sample was incubated at room temperature for 15 min.
• After incubation, the sample was centrifuged at a minimum speed of 12,000x*g* for 10 min.
• The supernatant was discarded by pipetting, and the pellet was resuspended in 1 mL of buffer RS by pipetting.
• The sample was centrifuged at a minimum speed of 12,000x*g* for 5 min, and the supernatant was discarded again by pipetting.
• The pellet was resuspended in 80 µL of buffer RL and briefly centrifuged.
• 20 µL of BugLysis (Kit 2) was added to the sample, followed by 1.4 µL of *β-mercaptoethanol* (Kit 2).
• The sample was vortexed for 15 s, and incubated at 37 °C for 30 min with shaking at 1000 rpm using a thermomixer.
• The sample was briefly centrifuged and 150 µL of buffer RP was added.
• 20 µL of Proteinase K (Kit 2) was added and vortexed for 15 s.
• The sample was incubated at 56 °C for 10 min, with shaking at 1000 rpm using a thermomixer.
• The sample was briefly centrifuged.
• 250 µL of buffer CS was added and vortexed for 15 s, then briefly centrifuged.
• 250 µL of buffer AB was added and vortexed for 15 s, then briefly centrifuged again to clear the lid.
• The lysate was then pipetted into a spin column, taking precautions to prevent the transfer of any particles that remain unresolved.
• The spin column was then centrifuged at a minimum speed of 12,000x*g* for 30 to 60 s.
• The column was then removed and placed in a new 2 mL collection tube.
• 400 µL of buffer WB was added and the spin column was centrifuged again at a minimum speed of 12,000x*g* for 30 to 60 s.
• The column was removed again and placed in a new 2 mL collection tube.
• 400 µL of 70% Ethanol was added and the spin column was centrifuged at a minimum speed of 12,000x*g* for 3 min.
• The column was carefully removed and placed in a 1.5 mL elution tube.
• 30 µL of deionized water, heated to 70 °C, was added to the column.
• The sample was incubated at room temperature for 1 min before centrifuging at a minimum speed of 12,000x*g* for 1 min.
• The column was discarded, the lid of the elution tube was closed, and the column was discarded.
• The eluted DNA was ready to be analyzed, or stored at −80 °C.

### Determination of quality and quantity of extracted DNA

Molecular identification techniques, including gel electrophoresis and the NanoDrop spectrophotometer (DeNovix DS-11), were utilized to evaluate the quality and quantity of the extracted DNA. Following the established protocol, a 1-liter volume of 1xTAE (Tris-acetate-EDTA) buffer was prepared by combining 25 mL of 40xTAE buffer with 975 mL of deionized water. A 1% agarose gel was prepared for electrophoresis by dissolving 0.3 g of agarose granules in 30 ml of TAE buffer using microwave-assisted techniques. The molten agarose solution was then cooled under flowing faucet water and poured into an electrophoresis cast, where it solidified for 20 min at room temperature. While waiting for the solidified gel, a 1:10,000 dilution of Diamond™ Nucleic Acid Dye was prepared by combining 0.1 L of the dye with 1000 µL of TAE buffer in a microcentrifuge tube. The dye was stored in a freezer at −20 °C for long-term storage. Afterwards, a 1 kb DNA ladder, DNA samples, 6x loading dye, and distilled water as negative control were meticulously inserted into the gel's designated wells. 30 min were spent with the gel electrophoresis apparatus operating at a constant voltage of 100 volts. After completion, the gel was examined under ultraviolet (UV) light using an Amersham Imager 680 (GE Healthcare, USA) to detect the DNA bands, which are indicative of DNA presence.

Prior to the quantitative examination, the NanoDrop spectrophotometer (DeNovix DS-11) was used to measure the concentration and purity of DNA samples based on the light absorption at 260 nm and 280 nm wavelengths. The purity of DNA was evaluated in accordance with the ratio of absorbance at A260/A280. Deionized water was used as blank during the absorbance readings.

### Library preparation using PCR amplification and sequencing

The amplification primers utilized were BITS-ACCTGCGGARGGATCA and B58S3-GAGATCCRTTGYTRAAAGTT in order to amplify the ITS1 region of fungi [11]. To facilitate inline barcoding, an additional 5 bases of inline barcode were added to the 5′ end of the primers [12]. Various samples were amplified utilizing different combinations of forward and reverse inline primers. For the preparation of the PCR reaction mixture, sterile PCR tubes were utilized within a biosafety enclosure to mitigate the risk of cross-contamination that could compromise the integrity of the results. Table 1 details the composition of the PCR reaction mixture. Subsequently, PCR amplification was conducted using the Eppendorf 5331 MasterCycle Gradient Thermal Cycler (Eppendorf, Germany) in accordance with PCR conditions displayed in Table 2.

**Table 1:** PCR reaction composition.

Component	Volume (µL)	Final concentration
Promega GoTaq Green Mastermix, 2X	10	1X
Forward primer, 1 µM	5	0.25 µM
Reverse primer, 1 µM	5	0.25 µM
DNA template	1.5	–
Total	21.5	–

**Table 2:** PCR condition and PCR reaction.

Component	Temperature	Time	Cycle
Initial denaturation	95 °C	2 min	1
Denaturation	95 °C	10 s	35
Annealing	48 °C	20 s	
Extension	72 °C	10 s	
Final extension	72 °C	1 min	1

After the PCR amplification was complete, the resulting PCR products were observed on an agarose gel containing 2% agarose, and SPRI Bead (Beckman Coulter, USA) was then used to purify them. Once the PCR products had been purified, a second index PCR was performed on the purified product. This step is intended to introduce Illumina-specific identifiers and complete the incorporation of the remaining Illumina adapter sequence into the DNA fragments. The index PCR was conducted in accordance with the PCR conditions detailed in Table 3.

**Table 3:** PCR conditions for index PCR.

Component	Temperature	Time	Cycle
Initial denaturation	95 °C	2 min	1
Denaturation	95 °C	10 s	8
Annealing	55 °C	10 s	
Extension	72 °C	10 s	
Final extension	72 °C	30 s	1

The barcoded amplicons obtained in the preceding stages were visualized using gel electrophoresis and were then aggregated based on the intensity of their respective bands. The aggregated amplicons were then subjected to DNA purification utilizing SPRI beads at a 0.8 X (v/v) ratio. The NEB Ultra II Library Preparation Kit (New England Biolabs, USA) was employed to process the purified pooled amplicons. The expected fragment size was confirmed, and the Promega Glomax reagent was used to quantify the libraries. In instances where some libraries exhibited a low yield, two distinct library pools with a ratio of samples were constructed to mitigate this. The final library pool's concentration was determined using Denovix high-sensitivity assay. The amplicon libraries and two controls were subsequently sequenced using a 2 × 150 bp configuration on an Illumina NovaSEQ6000 (Illumina, San Diego, CA) platform.

**Fig. 1:**
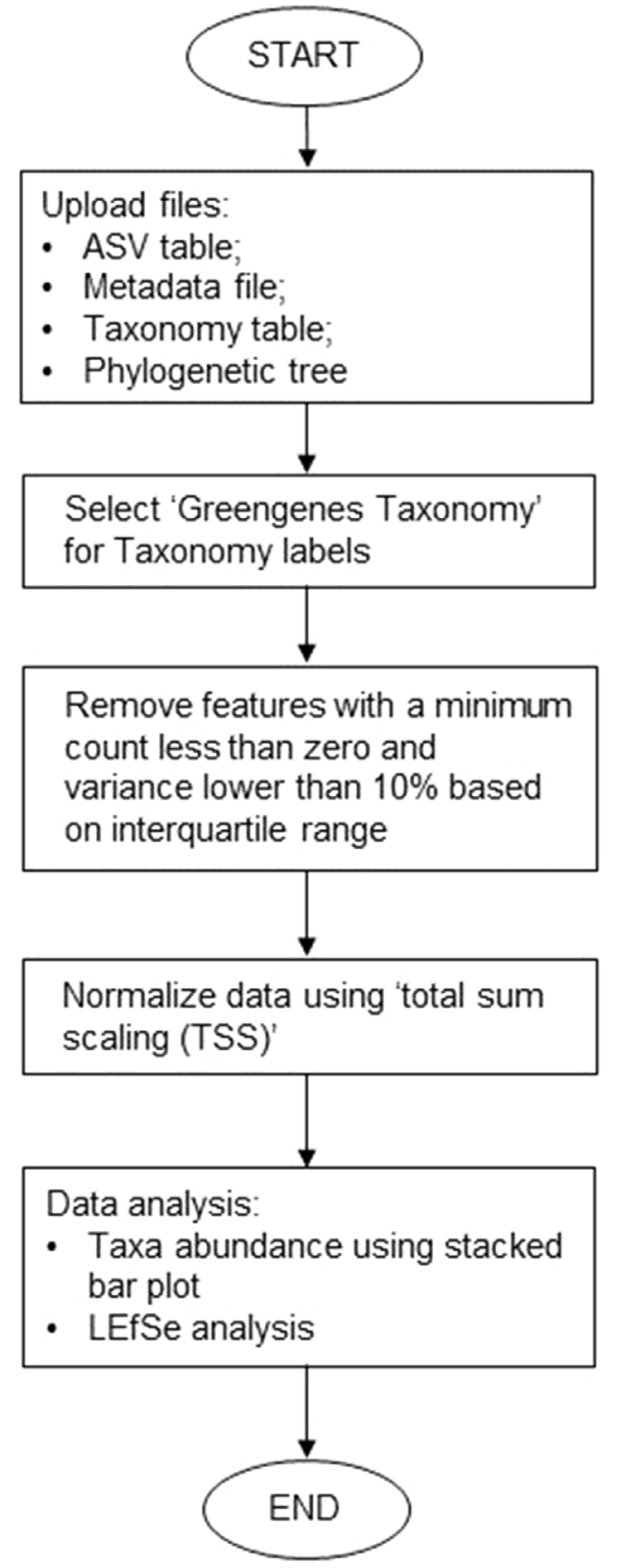
Workflow for data analysis using MicrobiomeAnalyst 2.0.

The unprocessed paired-end reads were overlapped using fastp v0.21 [13], followed by primer reduction using Cutadapt v1.18 [14]. The overlapping readings were incorporated into QIIME2 v.2022.22 [15] for subsequent denoising and construction of a count table using the DADA2 algorithm [16]. Amplicon sequence variants (ASVs) were classified using qiime2-feature-classifier, which was trained on the UNITE database [17]. The ASV table and taxonomic classification table were then meticulously formatted in accordance with MicrobiomeAnalyst's requirements so that they could be uploaded to the web server for data visualization (Fig. 1).

This study's mycobiome data were statistically analyzed utilizing MicrobiomeAnalyst 2.0 [18] with modifications [19], [20], [21]. Prior to analysis, features were filtered using predefined criteria, such as a minimum count threshold of zero and an interquartile range of 10%. Using total sum scaling (TSS), the filtered data were then normalized. A bar plot was constructed to visualize the taxonomic composition of the combined samples at the species level.

Statistically significant differences were identified using a significant threshold of p-value 0.05. Linear discriminant analysis Effect Size (LEfSe) was performed to identify the fungal taxa that are differentially abundant in CRC, polyps, and control groups. In addition, the genomic information of the opportunistic pathogens identified in the tumor samples was investigated further by searching the Bacterial and Viral Bioinformatics Resource Center (BV-BRC) (http://www.bv-brc.org). This analysis sought to establish the potential pathogenicity of the identified microorganisms.

### Method validation

The Ultra Deep Microbiome Prep kit (Molzym, Germany) was used to extract the microbial DNA from the biopsy samples. Molecular identification techniques, including gel electrophoresis and the NanoDrop spectrophotometer (DeNovix DS-11), were used to assess the purity and quantity of the extracted DNA during the DNA determination procedure. Gel electrophoresis was used to determine the presence of DNA bands and validate the efficacy of PCR amplification. Despite the inability to provide band diameters and intensities without the gel image, the presence of DNA was confirmed based on the experimental design and known results. The presence of DNA fragments within samples is indicated by the successful amplification of PCR products. The size of the PCR products targeting the ITS1 region ranged from 200 bp to 800 bp. In addition to gel electrophoresis, the concentration and purity of the extracted DNA were measured using a NanoDrop spectrophotometer. The NanoDrop spectrophotometer provided valuable information regarding the DNA's concentration and purity, further validating the extracted DNA's quality.

**Fig. 2:**
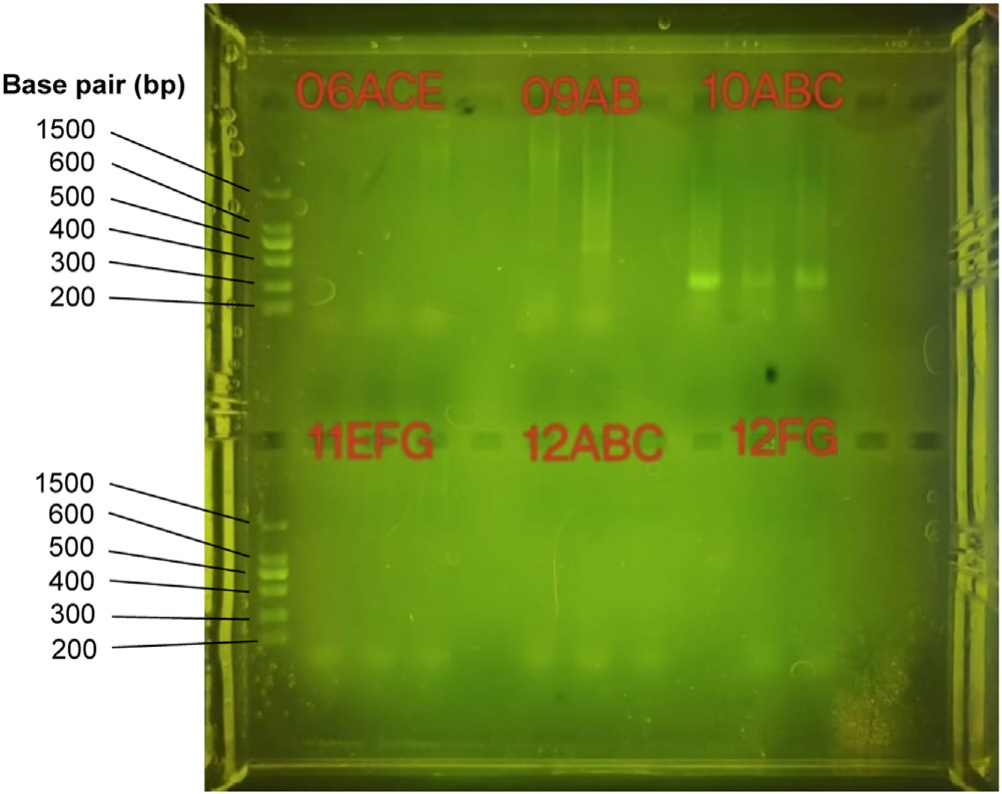
Optimized gel electrophoresis image of PCR products from biopsy samples (CRC: 06ACE – 10ABC, Polyps: 11EFG – 12ABC, Normal: 12FG).

PCR amplification was used to generate the DNA library for library preparation, with specific primers targeting ITS1 regions. Although precise information regarding band patterns and fragment sizes was challenging to be visible, the presence of PCR products indicates that amplification was 80% successful (Fig. 2). The observed pattern of scattering in the PCR products indicated the presence of non-specific or undesirable DNA fragments. Common causes of smearing include primer-dimer formation and the presence of impurities. The presence of scattering indicated that the PCR conditions must be optimized to increase specificity. Although the lack of gel images restricts the ability to provide specific visual evidence, the overall description emphasizes the presence of DNA and successful PCR amplification based on the experimental design and known results. The combination of gel electrophoresis and NanoDrop spectrophotometer analysis ensured the purity and suitability of the DNA samples for further analysis by validating the DNA determination process and library preparation. Furthermore, this study discovered that different sample types, either biopsy or stool, require specific PCR conditions for ITS1 fungal amplification. Therefore, the PCR conditions require to be optimized in order to ensure the visibility of PCR amplified fragments on the agarose gel before proceeding with amplicon sequencing.

**Fig. 3:**
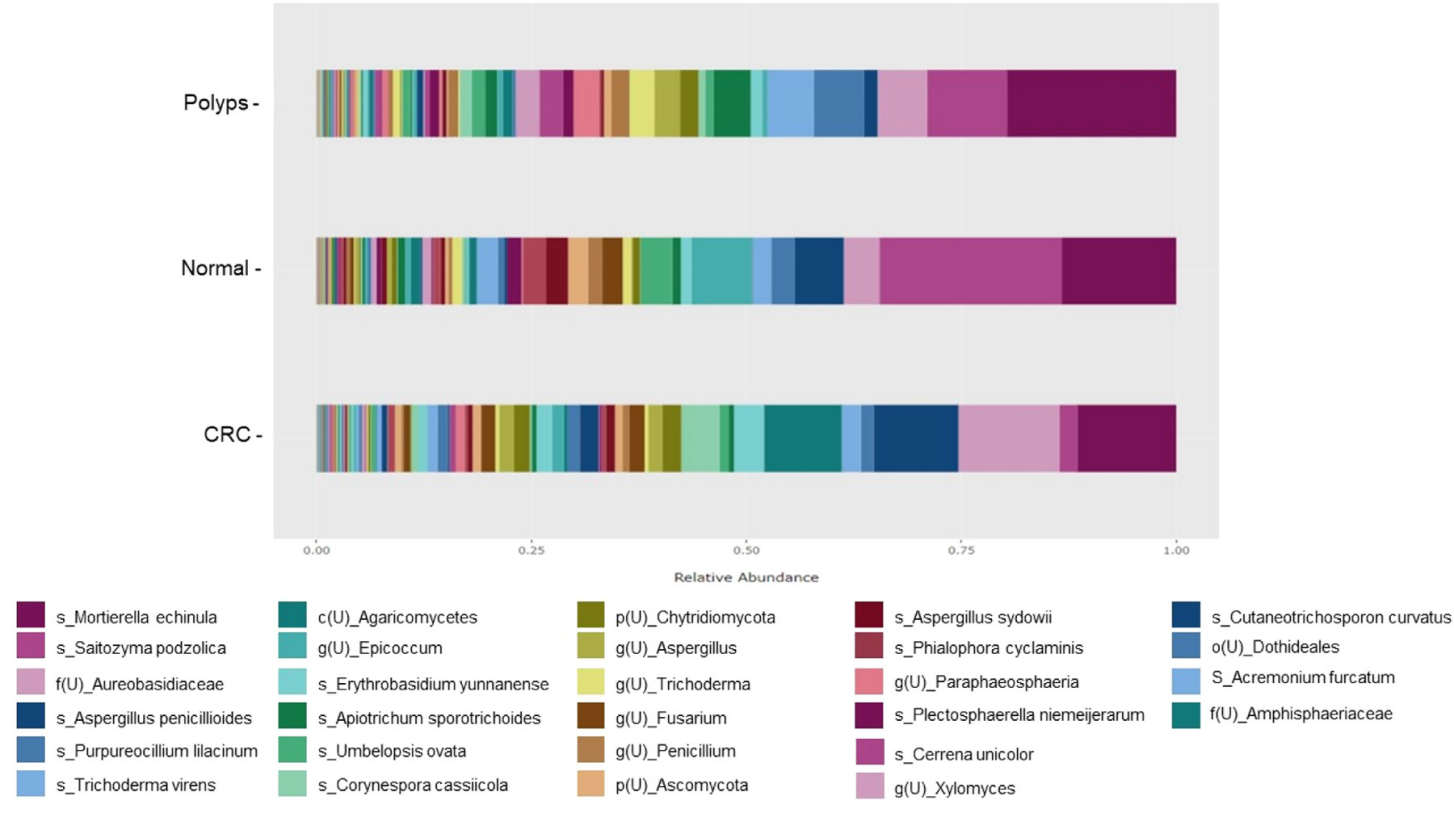
Relative abundance of fungal composition at the species level.

Amplicon sequencing was performed, yielding 6265,412 reads, and identifying 1364 ASVs in fungi. In various populations, the prevalence of these ASVs varied. Fig. 3 is a graphical representation of the relative abundance of fungal species at the species level in three clusters: patients with CRC, patients with polyps, and normal individuals.

In CRC patients, the top ten dominant taxa included *Aureobasidiaceae* at the family level with a relative abundance of 11.79%, *Mortierella echinula* (11.52%), *Aspergillus penicillioides* (9.85%), *Agaricomycetes* (9.02%) at the class level, *Corynespora cassiicola* (4.46%), *Erythrobasidium yunnanense* (3.41%), *Trichoderma virens* (2.23%), *Cutaneotrichosporon curvatus* (2.20%), *Chytridiomycota* (2.20%) at the phylum level, and *Saitozyma podzolica* (2.03%).

While in polyp patients, the top ten dominant taxa included *Mortierella echinula* (19.70%), *Saitozyma podzolica* (9.27%), *Purpureocillium lilacinum* (5.82%), *Aureobasidiaceae* (family level) (5.77%), *Trichoderma virens* (5.41%), *Apiotrichum sporotrichoid* (4.32%), *Paraphaeosphaeria* (genus level) (3.00%), *Trichoderma* (genus level) (2.96%), *Aspergillus* (genus level) (2.94%), and *Xylomyces* (genus level) (2.77%). It was suggested that *Mortierella echinula, Aureobasidiaceae* (family level) and *Trichoderma virens* have a potential association with both CRC and polyp patients, as they were abundantly presented in both clusters.

Among normal individuals, the top ten dominant taxa are *Saitozyma podzolica* (21.22%), *Mortierella echinula* (13.31%), *Epicoccum* (genus level) (6.98%), *Aspergillus penicillioides* (5.67%), *Aureobasidiaceae* (family level) (4.15%), *Umbelopsis ovata* (3.69%), *Purpureocillium lilacinum* (2.76%), *Phialophora cyclaminis* (2.70%), *Aspergillus sydowii* (2.53%), and *Acremonium furcatum* (2.44%).

These results emphasize the distinct composition of fungal species within each cluster and provide valuable insight into the fungal communities associated with CRC, polyps, and normal individuals. Utilization of the amplicon sequencing method enabled a comprehensive evaluation of fungal diversity, casting light on potential associations between certain fungal taxa and disease states. The presence of certain fungi, such as *Mortierella echinula* and *Saitozyma podzolica*, indicates their potential functions in the gastrointestinal ecosystem and their interactions with the host. To investigate the functional functions and implications of these fungi in the development and progression of CRC, additional research is required.

**Fig. 4:**
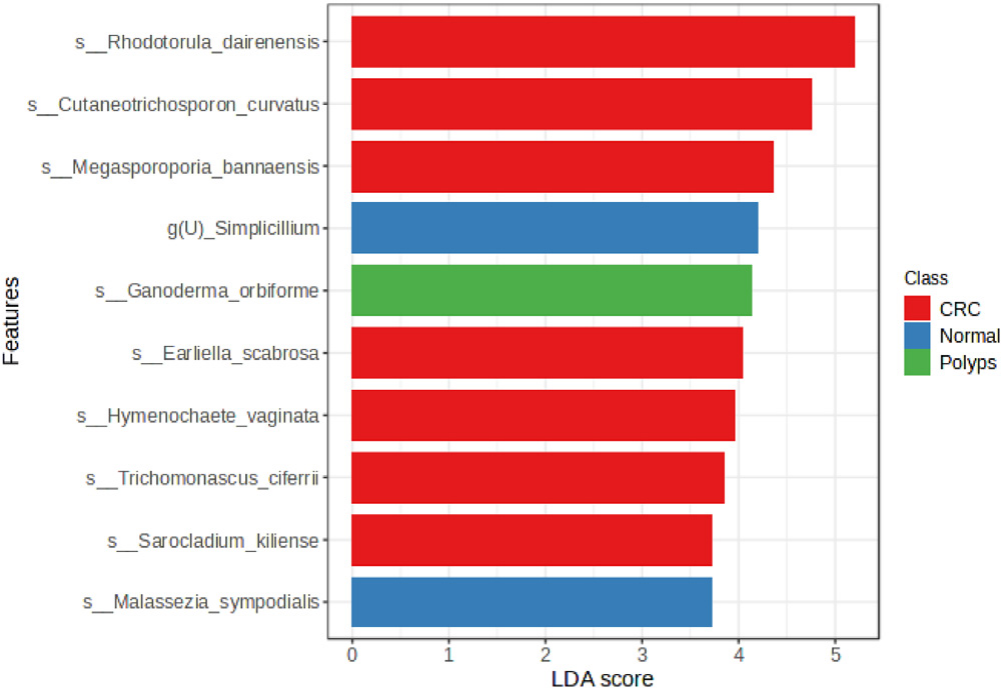
Linear discriminant analysis (LDA) score analysis showing 10 significant gut mycobiomes, at the species level.

Moreover, the LEfSe analysis was performed to identify fungal taxa with differential abundance between CRC, polyps, and normal clusters at the species level. LEfSe analysis is a useful tool to suggest the remarkable biomarkers between CRC, polyps, and normal patients. The LDA score assists in interpreting the degree of consistent variation in relative abundance between three clusters of analyzed fungal communities [22,23]. According to the results, as depicted in Fig. 4, ten fungal taxa exhibited statistically significant differences in abundance. *Rhodotorula dairenensis, Cutaneotrichosporon curvatus, Megasporoporia bannaensis, Earliella scabrosa, Hymenochaete vaginata, Trichomonascus ciferrii*, and *Sarocladium kiliense* were found to be significantly more abundant in CRC patients. These results imply that these fungal taxa may play a function in the CRC-associated microenvironment. Furthermore, *Ganoderma orbiforme* was found to be abundant in patients with polyps, suggesting its potential to be significant within the fungal communities associated with polyps development.

In contrast, two fungal taxa, *Simplicillium* and *Malassezia sympodialis*, exhibited significant differential abundance in normal individuals. The presence of these fungi in normal individuals suggests a possible association with a well-balanced gut microbiome and overall gastrointestinal health. Utilizing the LEfSe analysis enabled the identification of fungal taxa whose abundance varied significantly among clusters. The information was able to contribute to our knowledge of the specific fungal taxa associated with CRC and polyps, as well as the fungal taxa present in a normal gut mycobiome. These findings have significant ramifications for future research on the role of these fungal taxa in CRC pathogenesis and their potential as disease biomarkers or therapeutic targets.

### Conclusion

In conclusion, the methodology employed in this study, including the use of amplicon sequencing and bioinformatics analyses, has provided a robust method for examining the diversity and composition of the gut mycobiome in CRC patients, polyps, and normal individuals. The high-throughput sequencing method enabled the identification and profiling of all fungal taxa present in the biopsy samples. Furthermore, the implementation of LEfSE analysis allowed for the identification of specific fungal taxa whose abundance varied between CRC patients, polyp patients, and healthy individuals. These findings emphasize the potential function of these fungal taxa in CRC pathogenesis and imply their potential as disease biomarkers or therapeutic targets. Overall, the methodology employed in this study was effective in characterizing the gut mycobiome and investigating its potential relevance to CRC. Utilizing amplicon sequencing and bioinformatics analyses has provided a comprehensive and systematic method for elucidating the complex microbial community of the gastrointestinal. Further research based on this methodology can enhance our understanding of the intricate interactions between the gut mycobiome and CRC, hence paving the way for the future development of novel diagnostic and therapeutic strategies.

## Ethics statements

This study complied with all relevant ethical standards and guidelines to ensure the welfare and safety of the participants. The collection of samples for the ongoing study titled "Exploring Diet, Physical Activities, and the Host's Interaction with Colonic Mucosal Microbiota Population in Young Colorectal Cancer" has received registration and Ethical Clearance (UKM SPPI-BO02).

## CRediT authorship contribution statement

**Aisyah Yunus:** Data curation, Formal analysis, Investigation, Resources, Software, Validation, Visualization, Writing – original draft, Writing – review & editing. **Norfilza Mohd Mokhtar:** Conceptualization, Supervision, Validation. **Raja Affendi Raja Ali:** Conceptualization, Supervision, Validation. **Siti Maryam Ahmad Kendong:** Investigation, Resources, Writing – review & editing. **Hajar Fauzan Ahmad:** Conceptualization, Funding acquisition, Methodology, Software, Supervision, Validation, Writing – review & editing.

## Declaration of competing interest

The authors declare that they have no known competing financial interests or personal relationships that could have appeared to influence the work reported in this paper.

## Data Availability

Data will be made available on request.
